# Electrospun Tenofovir/Emtricitabine-Zein Nanofibers for Pre-Exposure Prophylaxis Platform against HIV through Vaginal Delivery

**DOI:** 10.1021/acsomega.5c08217

**Published:** 2025-11-12

**Authors:** Sinem Saar, Fatma Nur Tuğcu-Demiröz, Füsun Acartürk

**Affiliations:** Gazi University, Faculty of Pharmacy, Department of Pharmaceutical Technology, Etiler, Ankara 06330, Turkiye

## Abstract

Human immunodeficiency virus (HIV) remains a major global public health concern, particularly affecting vulnerable populations. Pre-exposure prophylaxis (PrEP) using tenofovir disoproxil fumarate (TDF) and emtricitabine (FTC) is a strategy recommended by the World Health Organization to prevent sexual transmission of HIV. Vaginal delivery systems based on nanofibers offer several advantages, including high surface area, mucoadhesiveness, and controlled drug release. This study aimed to develop and characterize TDF and FTC-loaded zein-based electrospun nanofibers for vaginal PrEP. Electrospun hybrid nanofibers were produced using zein in combination with mucoadhesive polymers such as polyvinylpyrrolidone (PVP) and poly­(ethylene oxide) (PEO). The nanofibers were characterized by Fourier transform infrared spectroscopy (FTIR), differential scanning calorimetry (DSC), and scanning electron microscopy (SEM). Mechanical strength and mucoadhesive properties were evaluated for vaginal use. In vitro release of TDF and FTC from the nanofibers was also investigated. The ZK7-TF formulation showed 898.048 ± 300.838 nm uniform diameters and porosity 60.03%. Mucoadhesion work reached 0.121 ± 0.014 mJ·cm^–2^, while mechanical integrity was supported by 1.510 ± 0.501 MPa tensile strength and 2.617 ± 0.977% elongation at break values. Hydrophilic properties of TDF and FTC-loaded formulations were evidenced by contact angles of <90°. The release studies showed controlled release of TDF and FTC for 24 h. Zein-based hybrid nanofibers incorporating mucoadhesive polymers represent a promising drug delivery system for the vaginal delivery of antiretroviral drugs as an effective and patient-compliant strategy for HIV prevention. This study provides a comprehensive formulation approach, combining zein with poly­(ethylene oxide) and polyvinylpyrrolidone to fine-tune nanofiber morphology, mechanical strength, mucoadhesion, and drug release properties for vaginal delivery.

## Graphical abstract

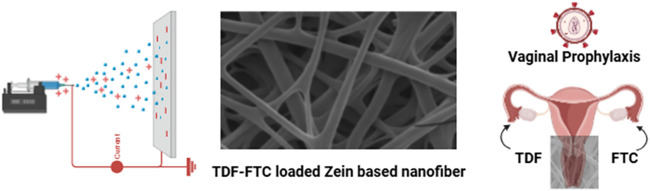

## Introduction

Acquired immunodeficiency syndrome (AIDS) is an immune system disorder caused by the human immunodeficiency virus (HIV). HIV suppresses the immune system by destroying cells that play a role in the body’s defense mechanisms.^1^ The virus continues to spread globally and is a major public health issue. The disease is increasingly prevalent particularly among women, homosexual individuals and migrants.^2,3^ Tenofovir disoproxil fumarate (TDF) and emtricitabine (FTC) based oral pre-exposure prophylaxis is recommended by the World Health Organization as an effective strategy to prevent sexual HIV transmission.^4^ The combination of TDF and FTC is an FDA-approved oral pre-exposure prophylaxis (PrEP) regimen, available under the name Truvada.^5^ However, patient compliance with oral administration is one of the most significant factors limiting the treatment effectiveness. Factors such as the difficulties of daily oral dosing, gastrointestinal side effects, systemic exposure, and lifestyle incompatibility can reduce patient compliance. Therefore, there is a need to develop alternative drug delivery systems that increase patient compliance. Adherence is considered the main factor limiting the efficacy of orally administered TDF and FTC; however, other underlying factors may also play a significant role in their clinical effectiveness.^6^ Various dosage forms containing TDF and/or FTC have been developed, including gels, vaginal rings, films, tablets, and nanoparticles.^4,7–9^ However, these dosage forms have several disadvantages that may negatively impact patient compliance, such as frequency of application, difficulty in use, leakage, or local irritation. Microbicides are products developed to provide protection against HIV and sexually transmitted infections, which are especially needed in women of reproductive age due to the high risk of transmission.^10^ Dosage forms developed as microbicides include gels, films, vaginal rings, and vaginal tablets, which are administered topically.^4,8,11,12^

Nanofibers can be fabricated using various techniques, including phase separation, self-assembly, template synthesis, and electrospinning.^13,14^ Electrospinning is a simple, useful, and versatile method for producing polymeric nanofibers and enables the production of uniform, continuous, and porous nanofibers from polymeric solutions using electrostatic force.^15^ Nanofibers are fiber-shaped one-dimensional nanomaterials with diameters ranging from tens to hundreds of nanometers.^16,17^ Electrospinning effectively overcomes the complexity, poor reproducibility, and toxicity issues of other fiber production methods, while enabling consistent nanofiber fabrication with desirable structural and functional properties.^18^

Nanofibers have several advantages in vaginal applications such as high surface area, solubility, stability, flexibility, better mechanical characteristics, ease of surface modification, excellent drug loading efficiency and controlled drug release.^19^ Nanofibers are used in a variety of therapeutic applications with different structural properties depending on the type of polymer. Nanofibers produced by electrospinning combine the advantages of natural and synthetic polymers, offering enhanced mechanical and biological properties in a simple and cost-effective manner.^20^ It is an effective approach frequently used to improve the functional properties of nanofiber systems developed by using biopolymers with different components.^21^ By combining the properties of different polymers synergistically, it is possible to optimize multidimensional properties such as chemical stability, structural integrity, mechanical strength, morphological control, and biocompatibility.

Zein, a protein derived from corn, has been studied for vaginal applications for its nontoxicity, biodegradability, and stability over a wide pH range. Studies involving zein-based systems loaded with various agents for administration via different routes such as buccal, vaginal, and transdermal have been reported in the literature.^22,23^ Notario-Pérez and colleagues developed vaginal film formulations containing zein and HPMC. This study aimed to provide controlled release of TFV to prevent sexually transmitted HIV infection through vaginal film formulations. Polyvinylpyrrolidone (PVP) and poly­(ethylene oxide) (PEO) are used in vaginal drug delivery systems due to their highly water-soluble, nontoxic, mucoadhesive, biocompatible, and biodegradable properties.^19^ These polymer combinations enable enhanced mucosal interactions by optimizing both the mechanical properties and drug release characteristics of the formulation.

Electrospun fiber systems for vaginal administration of tenofovir and emtricitabine are expected to exhibit promising drug release behavior and improved local drug concentrations compared with oral formulations. In this context, the coloading of tenofovir disoproxil fumarate (TDF) and emtricitabine (FTC) into zein-based nanofibers is hypothesized to provide both enhanced antiretroviral efficacy and the inherent advantages of vaginal delivery. The aim of the study is to develop a novel zein-based hybrid nanofiber formulation loaded with TDF and FTC for potential vaginal pre-exposure prophylaxis (PrEP) against HIV. The incorporation of zein with PVP and PEO resulted in nanofibers with favorable mechanical, mucoadhesive, and physicochemical characteristics. Moreover, the ability to modulate drug release profiles through this nanofiber system offers a promising alternative to conventional gel- or ring-based PrEP formulations. To the best of our knowledge, this is among the first studies to explore zein nanofibers for the combined delivery of TDF and FTC via the vaginal route, positioning them as a promising candidate for innovative HIV prevention strategies.

## Materials and Methods

### Materials

Tenofovir disoproxil fumarate (TDF) was kindly provided by Gen İlaç Türkiye, and emtricitabine (FTC) was generously supplied by İlko Pharmaceuticals (Türkiye). Zein was purchased from Sigma-Aldrich. PEO (Polyox, WSR 205, MW: 600 kDa) and PEO (Polyox, WSR-N80, MW: 200 kDa) were kindly donated by FMC BioPolymer. PVP K90 was a gift from BASF. Other chemicals used were of analytical grade.

### Electrospinning Process

Nanofiber formulations were manufactured by the electrospinning method. The presence of numerous neutral amino acid residues in the structure of zein renders it insoluble in aqueous media; however, it becomes soluble in specific concentrations of ethanol, anionic surfactants, or alkaline solutions.^24^ Polymer solutions containing zein were prepared using an 8:2 ethanol:distilled water. Due to the low mechanical strength and mucoadhesive properties of zein, its combinations with PEO and PVP polymers have been investigated to enhance characteristics in the formulations (Figure 1). PEO with two different molecular weights was used for the formulations. Low molecular weight PEO (Mw: 200 kDa) was coded as N and high molecular weight (Mw: 600 kDa) was coded as W. In the formulation design, PEO WSR 205 was fixed at 1% to avoid excessive viscosity that impeded fiber formation. PEO N80 and PVP K90 were incorporated up to 5% based on previous reports.^25–30^ PVP concentrations were further varied, as this polymer demonstrated favorable effects on the mechanical strength and mucoadhesion. Additionally, different zein concentrations were investigated to evaluate their influence on the nanofiber morphology and properties. All formulations are listed in Table 1. Throughout all electrospinning processes, the collector rotation speed was kept constant at 100 rpm to ensure uniform fiber deposition and structural consistency among the formulations.

**Figure 1:**
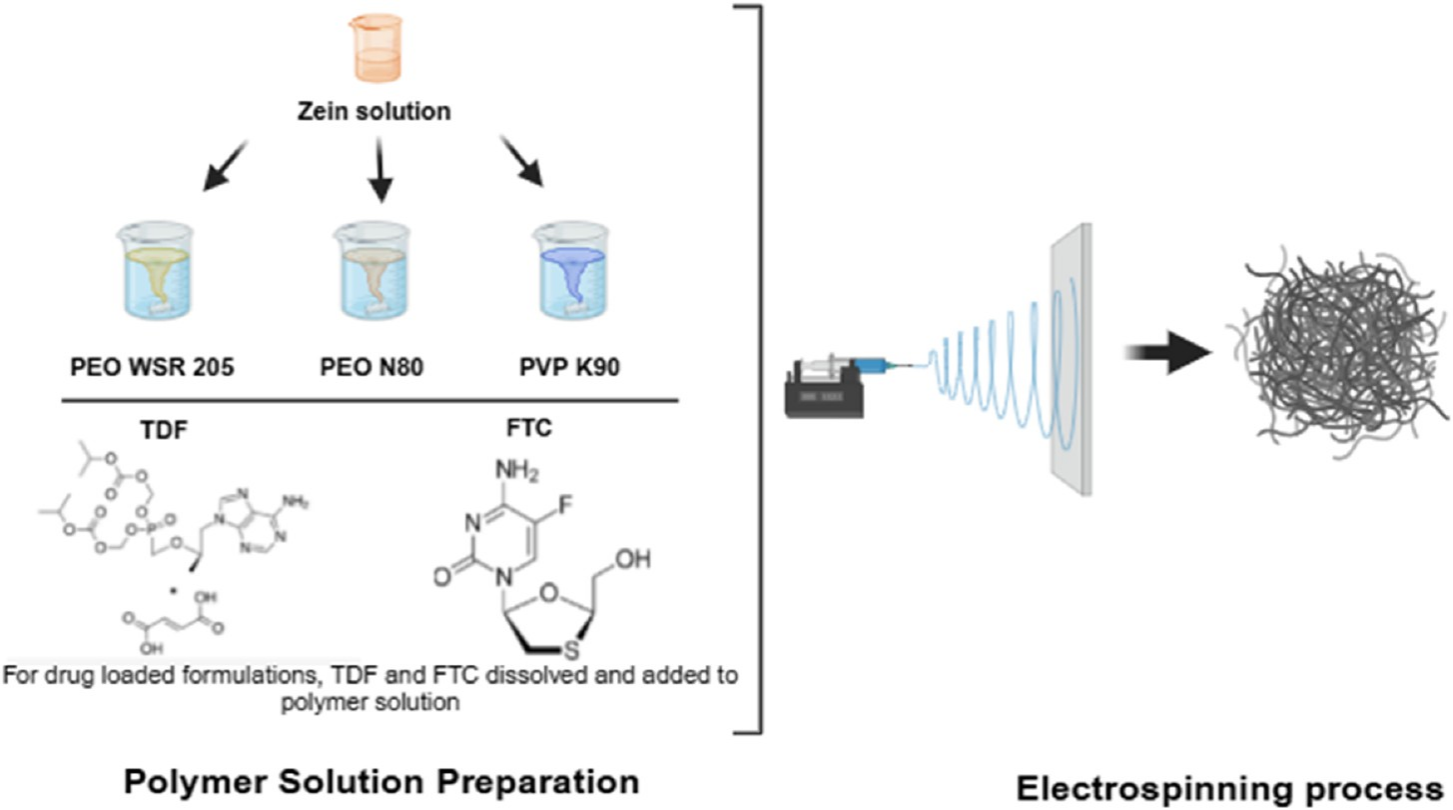
Schematic illustration of the preparation of zein-based nanofibers by electrospinning. (This figure was created with biorender.com).

**Table 1:** Codes and Composition of Zein-Based Vaginal Nanofiber Formulations

codes	zein (%)	PEO WSR 205 (%)	PEO N80 (%)	PVP K90 (%)
Z1	20			
Z2	30			
ZW0		1		
ZW1	2.5	1		
ZW2	5	1		
ZW3	10	1		
ZW4	20	1		
ZN0			5	
ZN1	2.5		5	
ZN2	5		5	
ZN3	10		5	
ZN4	20		5	
ZN5	30		5	
ZK0				5
ZK1	2.5			5
ZK2	5			5
ZK3	10			5
ZK4	20			5
ZK5	30			5
ZK6				10
ZK7	5			10
ZK8	10			10

Zein was dissolved in ethanol:water (8:2) and combined with mucoadhesive polymers (PEO WSR 205, PEO N80, PVP K90). For drug-loaded formulations, TDF and FTC were dissolved and incorporated into the polymer solution prior to electrospinning.

TDF and FTC were incorporated into the optimized nanofiber formulations to develop vaginal delivery systems intended for effective pre-exposure prophylaxis (PrEP) against HIV. For this purpose, TDF and FTC were dissolved in the polymer solvent system and added to the polymer mixture to be used (Table 2). The mixture was mixed in a magnetic stirrer until a homogeneous mixture was obtained. Before the electrospinning process, polymer solutions were kept for 1 min in an ultrasonic bath to remove air bubbles. All formulations were prepared by using a fixed collector rotation speed of 100 rpm. Since the protective vaginal dose of TDF/FTC has not yet been determined in humans, the rationale used in the current study was established by reviewing literatures. It has been shown that 30–150 μg of TDF in a thermosensitive gel formulation containing poly­(lactic-*co*-glycolic acid) nanoparticles can provide complete protection against intravaginal challenge with HIV in humanized mice.^31^ Nunes and colleagues examined the efficacy of intravaginally administered fibers in an animal model. They stated that the vaginal doses of TDF (0.07 mg) and FTC (0.05 mg) were chosen based on previous studies demonstrating efficacy in preventing vaginal HIV-1 transmission in humanized mice. They suggested that the fibers developed could be an alternative or complement to current oral PrEP, which requires daily administration of TDF/FTC.^6^ In our study, the vaginal doses were adjusted to contain 0.9% TDF and 0.6% FTC based on Truvada. Moreover, high drug loading in electrospinning may lead to poor distribution of the active ingredient within the polymer matrix, adversely affecting fiber morphology, drug dissolution, and release profiles. Therefore, the selected drug ratios were intended to ensure both sufficient therapeutic levels and uniform dispersion within the nanofibers, ultimately supporting consistent and controlled drug release.

**Table 2:** Codes and Composition of TDF (0.9%) and FTC (0.6%)-Loaded Zein-Based Vaginal Nanofiber Formulations

codes	zein (%)	PEO N80 (%)	PVP K90 (%)
Z2-TF	30		
ZN3-TF	10	5	
ZK3-TF	10		5
ZK7-TF	5		10
ZK8-TF	10		10

The electrospinning process parameters of the formulations are listed in Table 3. The parameters of the electrospinning method varied according to the polymer concentration. During the electrospinning process, nozzle clogging was occasionally observed, which was mainly attributed to the viscosity of zein solutions, solvent evaporation, and the applied flow rate. Such nozzle clogging-induced instabilities represent a critical factor influencing fiber morphology, structural uniformity, and ultimately the reproducibility of zein-based nanofiber formulations.^32^ To address this, the electrospinning process was continuously monitored, and adjustments in flow rate, applied voltage, and needle–collector distance were implemented to restore a stable jet.

**Table 3:** Production Parameters of Zein-Based Vaginal Nanofiber Formulations

formulation code	voltage (kV)	feed rate (mL/h)	distance (mm)
Z1	20.5	0.3	80
Z2	20.5	0.4	100
ZW0	6.5	0.6	200
ZW1	8	1	200
ZW2	5.5	1.5	190
ZW3	7	1	230
ZW4	7	2	225
ZN0	16	1	120
ZN1	16.5	1.5	105
ZN2	13.5	1.8	120
ZN3	12.5	0.5	115
ZN4	13	2.5	120
ZN5	13.5	1.5	130
ZK0	17.5	2	120
ZK1	16.5	2.5	110
ZK2	17	1.5	120
ZK3	16.5	2.4	120
ZK4	17	2	125
ZK5	16.5	0.5	165
ZK6	16.5	7.5	130
ZK7	16.5	1	155
ZK8	16.5	2.2	155

Process parameters changed with the addition of TDF and FTC to the polymer solutions. The electrospinning process parameters of the blank and TDF/FTC-loaded formulations are given in Tables 3 and 4, respectively.

**Table 4:** Electrospinning Process Parameters of TDF and FTC-loaded Zein-Based Vaginal Nanofiber Formulations

formulation code	voltage (kV)	feed rate (mL/h)	distance (mm)
Z2-TF	20.5	0.5	90
ZN3-TF	20	1.5	12
ZK3-TF	19.5	2	15
ZK7-TF	19	1.8	16
ZK8-TF	20	1.5	12

### Characterization Studies of Polymer Mixtures

Viscosity measurements of polymer mixtures were performed with a stress-controlled cone and plate rheometer (Brookfield, DV-III Rheometer with spindle type CPE-41). All the samples were measured at room temperature. Conductivity measurements of the polymer mixtures were carried out using a conductometer. The pendant observation drop method was used to measure the surface tension.^33^ The surface tension of the polymer mixtures was determined by forming drops at the tip of the needle and analyzing them using the pendant drop method (Attension-Theta Lite, Biolin Scientific, Finland). The Young–Laplace equation was used to calculate the surface tension of polymer mixtures.^34^ Measurements were repeated three times for each polymer mixture.

### Differential Scanning Calorimetry Analysis (DSC)

DSC (Shimadzu, DSC-60, Japan) analysis was carried out on the polymers, polymer/drug mixtures (1:1), TDF, FTC, and the vaginal nanofibers. Approximately 2 mg of each sample was compressed into an aluminum pan. The samples were heated from 25 to 250 °C at a heating rate of 10 °C/min under a nitrogen atmosphere.

### Fourier Transform Infrared Spectroscopy

Fourier transform infrared (FTIR) spectroscopy analyses of the TDF, FTC, zein, PEO, PVP, and optimum formulation were performed using FTIR (PerkinElmer, Spectrum 400 FTIR). Each spectrum was recorded between 600 and 4000 cm^–1^.

### Morphological Characterization

Morphological characterization of all formulations was performed using Scanning Electron Microscopy (Quanta 400, FEI). SEM is a useful method for evaluating the effects of the electrospinning process parameters on nanofibers. The nanofibers were coated with gold and palladium for SEM analyses. The images were taken in different parts of the nanofibers in 1000, 5000, and 10,000 magnification. Average fiber diameters were determined by measuring fibers randomly selected from SEM images using ImageJ (National Institutes of Health, Bethesda). The porosity of the vaginal nanofiber formulations was calculated from SEM images using ImageJ software (Version 1.54, National Institutes of Health). In this method, nanofibers were digitally processed by converting fiber regions to black and pore regions to white. The percentage of the white area relative to the total image area was defined as the porosity (%), representing the void fraction within the nanofiber matrix.^16^

### Mechanical Characterization

Mechanical properties of vaginal nanofiber formulations were carried out using a Texture Analyzer (TA.XT. PlusTexture Analyzer, Stable Micro Systems, UK) equipped with a mini tensile grip. Nanofibers were cut into a rectangular shape (3 × 1 cm) and used in the apparatus. The tensile tests were conducted at a strain rate of 5 mm/min. Tensile strength (MPa) and elongation at break (%) measurements were calculated from the strain–stress graphs. Each measurement was performed in triplicate at room temperature, and the mean ± SD values were reported.

### Contact Angle Measurements

The contact angle measurements of vaginal nanofiber formulations were carried out by an Attension Theta Lite optical tensiometer. The vaginal nanofiber formulations were stretched on a convex sample holder. Measurements were carried out by dropping 5 μL of distilled water onto the nanofiber surface. A contact angle of 0–90° indicates good wettability, and a contact angle of 90–180° indicates hydrophobicity.^35^

### Mucoadhesion Studies of Vaginal Nanofiber Formulations

Mucoadhesive properties of formulations were analyzed with a texture analyzer TA-XT. Ex vivo mucoadhesion studies were conducted using cow vaginal tissue. The tissue taken from the cow was stored at −20 °C. Sections of appropriate size for mucoadhesion were taken from the tissue that was removed and thawed before the experiment. The mucoadhesion study was carried out using previously approved test conditions.^36^ The force required for the separation of the formulations from the mucosal surfaces was measured to evaluate mucoadhesive strength. The work of mucoadhesion was calculated from the obtained data using the formula (mJ/cm^2^) = AUC/(π*r* ^2^).^37,38^

### In Vitro Permeation Studies

Permeability studies for the optimum formulations were performed using Franz-type diffusion cells with a cross-sectional area of 1 cm^2^. A dialysis membrane (Sigma) permeable to molecules smaller than 12,000 Da was used in the experiments. The membrane used was placed in a horizontal diffusion cell. The stirred 2.5 mL receptor phase containing the simulated vaginal fluid (SVF) (pH 4.2) was thermostated at 37 °C. 2.5 mL portion of SVF was placed in the receptor cell. This system was placed in a 37 °C water bath. Formulations were placed in the donor compartment, and 2.5 mL samples were periodically taken from the receptor phase and filled with the same volume of fresh SVF. All in vitro release studies for both TDF and FTC were performed under sink conditions. Quantification of TDF and FTC in the formulations was performed by using high-performance liquid chromatography (HPLC).

### Quantitation of TDF and FTC

The quantification of TDF and FTC in SVF was carried out using a validated HPLC method.^4,6^ Validation parameters included linearity, accuracy, precision (repeatability and intermediate precision), sensitivity (limit of detection and limit of quantitation), selectivity, robustness, and stability. For the quantification of TDF, an isocratic HPLC method was employed using an XSelect Extend C18 column (250 × 4.6 mm). The mobile phase consisted of a mixture of 1 mM phosphate buffer (pH 5.0) and acetonitrile in a ratio of 70:30 (v/v). The flow rate was set to 1.5 mL/min; the column temperature was maintained at 30 °C, and the detection wavelength was 260 nm. TDF showed a retention time of approximately 5.6 min. For the FTC, a gradient elution method was necessary to achieve adequate separation and resolution. The analysis was carried out using a C18 column (250 × 4.6 mm) at a flow rate of 1.0 mL/min and a column temperature of 35 °C. The mobile phase was composed of 1 mM phosphate buffer (pH 5.0) and acetonitrile, and the gradient profile was optimized to begin with a high aqueous phase and gradually increase the acetonitrile content. FTC was detected at 280 nm, with a retention time of approximately 4.7 min. Both methods demonstrated high specificity with no interfering peaks detected from blank matrices or formulation excipients. The retention times were consistent and the peak shapes were symmetrical. The limits of detection (LOD) and quantitation (LOQ) for TDF were 0.0140 and 0.0425 μg/mL, respectively; for FTC, the LOD and LOQ were 0.0260 and 0.0787 μg/mL, respectively, indicating high method sensitivity.

### Statistical Analysis

Experiments were performed in triplicate. Data are presented as mean ± standard deviation (SD). Multiple group comparisons were performed by one-way ANOVA with Tukey’s HSD posthoc using Prism 5 software. A *p* value < 0.05 was considered statistically significant.

## Result

Zein-based electrospun nanofibers were fabricated as vaginal drug delivery systems for antiretroviral agents (TDF and FTC). In this study, different ratios and types of mucoadhesive polymers, PVP and PEO, were used to prepare vaginal nanofibers in order to increase the retention time of the formulations in the vagina. Zein/PVP and zein/PEO-based hybrid nanofiber formulations were prepared by the electrospinning method, and their process parameters were optimized to achieve a smooth and bead-free fiber morphology. Following the comprehensive characterization of blank nanofiber formulations, the most promising candidates were selected and further evaluated after the incorporation of TDF and FTC for their suitability in vaginal drug delivery applications.

### Characterization Results of Polymer Mixtures

Polymer solutions were characterized by viscosity, conductivity, and surface tension measurements for the electrospinning process (Table 5). Results showed that formulations with higher viscosity and intermediate conductivity produced more homogeneous fibers, while lower viscosity resulted in bead formation and brittle structures. These properties influence nanofiber morphology and play a critical role in the performance of the formulations for vaginal drug delivery applications. The conductivity, viscosity, and surface tension values generally increased with the addition of TDF and FTC to the zein-based vaginal nanofiber formulations, as seen in Table 6.

**Table 5:** Viscosity, Conductivity, and Surface Tension Values of Polymer Solutions for Vaginal Nanofiber Formulations

formulation code	viscosity (cP·s)	conductivity (μS/cm)	surface tension (mN m^–1^)
Z1	44 ± 0	1238.33 ± 1.52	22.66 ± 0.05
Z2	309.33 ± 1.15	1312.33 ± 1.52	22.13 ± 0.32
ZW0	237 ± 5	46.64 ± 0.50	45.20 ± 0.72
ZW1	320.33 ± 11.15	318.77 ± 4.90	47.48 ± 0.90
ZW2	56.66 ± 1.15	638.50 ± 0.45	23.80 ± 0.03
ZW3	95 ± 0	887.10 ± 2.28	23.70 ± 0.10
ZW4	175.66 ± 1.15	1208 ± 2	23.02 ± 0.06
ZN0	326.33 ± 14.5	23.99 ± 0.29	31.30 ± 0.24
ZN1	354.33 ± 16.65	146.6 ± 6.36	29.63 ± 0.15
ZN2	329 ± 0	501.83 ± 1.15	23.68 ± 0.04
ZN3	384.33 ± 1.15	866.70 ± 1.35	23.98 ± 0.03
ZN4	431.33 ± 1.15	1208.33 ± 0.57	23.47 ± 0.02
ZN5	572.33 ± 1.15	1315.66 ± 2.08	23.23 ± 0.04
ZK0	338.67 ± 28.91	42.76 ± 2.96	29.25 ± 0.11
ZK1	247.33 ± 41	269.2 ± 11.79	28.79 ± 0.16
ZK2	485.66 ± 1.15	609.77 ± 0.57	27.23 ± 5.93
ZK3	536.33 ± 4.16	815.93 ± 3.05	23.42 ± 0.09
ZK4	952.66 ± 3.05	1026.66 ± 1.52	23.23 ± 0.10
ZK5	953.33 ± 6.42	1164 ± 4.58	23.6 ± 0.6
ZK6	402.33 ± 35.23	23.88 ± 1.33	27.14 ± 0.67
ZK7	470 ± 0	465.10 ± 4.11	23.05 ± 0.18
ZK8	525.67 ± 1.15	751.73 ± 10.96	22.99 ± 0.47

**Table 6:** Characterization Results of Polymer Solutions of Zein-Based Vaginal Nanofiber Formulations Containing TDF and FTC

formulation code	viscosity (cP·s)	conductivity (μS/cm)	surface tension (mN m^–1^)
Z2-TF	362 ± 11	1199 ± 4	27.35 ± 0.07
ZN3-TF	366 ± 11	741.33 ± 2.27	27.57 ± 0.29
ZK3-TF	546 ± 2	766.47 ± 2.55	28.80 ± 0.11
ZK7-TF	442 ± 18	616 ± 8	28.18 ± 0.11
ZK8-TF	543.33 ± 19.50	887.37 ± 11.50	28.73 ± 0.06

### Morphological Characterization of Formulations

The morphology of the zein hybrid nanofibers was evaluated to determine the effects of the electrospinning parameters on fiber formation (Figure 2). Morphological characterization was performed only for the selected formulations, as the remaining formulations exhibited brittle or nonuniform structures unsuitable for SEM analysis. The diameter of the hybrid nanofibers is affected by the electrospinning process according to polymer type and concentration. As the polymer concentration increased, the fiber diameters generally became larger and more uniform, while low concentrations resulted in thinner fibers with occasional bead formation. The presence of hydrophilic polymers (PVP or PEO) increased the solution viscosity and improved fiber formation, reducing bead defects. The nanofibers exhibited a smooth and uniform structure without bead formation, indicating the optimized processing conditions.

**Figure 2:**
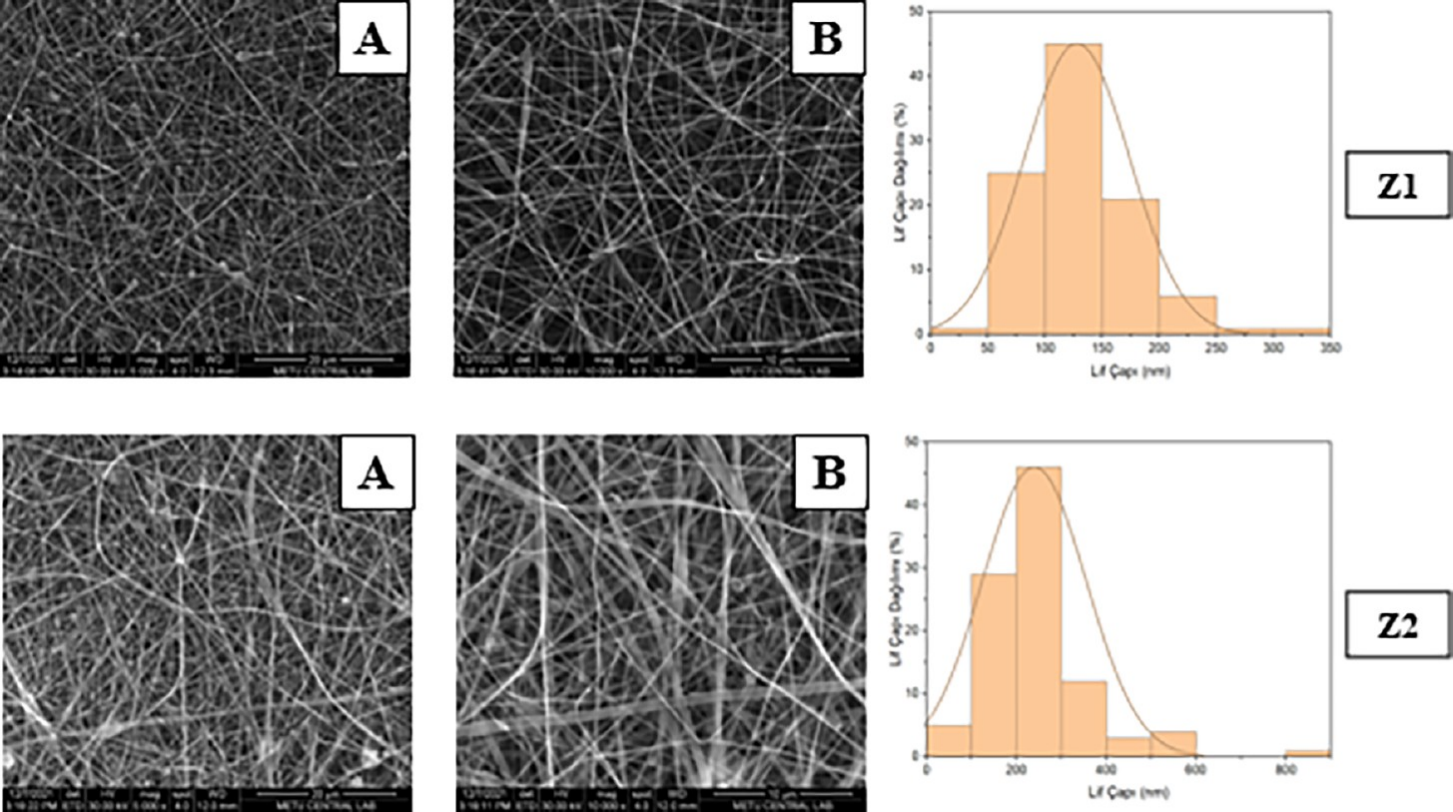
SEM images and fiber diameter distributions of Zein (Z series) nanofibers (A: 5000×, B: 10,000×).

The fiber diameter varied significantly depending on the zein, PEO, and PVP polymer ratios, and the viscosity, conductivity, and surface tension parameters of the solution (Table 7). Fiber diameters in nanometer dimensions provide properties such as increased specific surface area and high porosity in the fibers.

**Table 7:** Fiber Diameters and Porosity of Zein-Based Vaginal Nanofiber Formulations

formulation code	fiber diameter (nm)	porosity (%)
Z1	127.896 ± 46.212	62.16
Z2	239.684 ± 115.792	55.78
ZW2	477.617 ± 110.466	69.62
ZW3	1658.242 ± 695.677	60.54
ZW4	2553.760 ± 1158.028	42.32
ZN2	297.843 ± 111.473	51.26
ZN3	527.214 ± 167.178	66.03
ZN4	1098.421 ± 468.775	60.95
ZN5	2538.829 ± 1356.231	51.45
ZK1	341.141 ± 124.654	71.78
ZK3	581.682 ± 268.433	58.52
ZK4	1836.530 ± 933.900	56.06
ZK5	2285.209 ± 1489.735	53.98
ZK7	477.611 ± 176.885	64.18
ZK8	977.988 ± 500.015	59.58

### DSC Analysis Results

DSC analysis of TDF, FTC, polymers, and formulation are shown in Figure 7. The characteristic peak of TDF was found at 118.29 °C. Literature data indicate two distinct melting peaks for TDF: the first between 112 and 114 °C corresponding to the α-polymorph, and the second between 117 and 118 °C corresponding to the β-polymorph (Table 8).^39,40^

**Table 8:** Fiber Diameters and Porosity of Zein-Based Vaginal Nanofiber Formulations Containing TDF and FTC

formulation code	fiber diameter (nm)	porosity (%)
Z2-TF	622.2 ± 224.6	49.93
ZN3-TF	930.6 ± 288.7	50.35
ZK3-TF	791.0 ± 289.8	59.53
ZK7-TF	898.1 ± 300.8	60.03
ZK8-TF	1312.1 ± 402.4	43.77

The DSC thermogram of FTC indicates that the compound melted prior to thermal degradation. The initial phase of FTC was thermal degradation involving both intermolecular and intramolecular oxidation processes. The first peak of FTC is characterized by an endothermic peak, suggesting that this transition primarily corresponds to a thermal melting process. The second peak presents an exothermic peak, indicating an oxidative degradation mechanism. Fathima et al. reported a sharp endothermic peak at 153.40 °C in the DSC thermogram of pure FTC, corresponding to its melting point, followed by an exothermic peak at 256.81 °C, which reflects the onset of thermal decomposition due to oxidative processes.^41^ TDF and FTC were found to be consistent with the melting point range reported in the literature.

Zein does not exhibit a sharp melting point due to its amorphous nature. Thermal degradation of zein starts at 25 °C. Hu et al. reported that DSC of pure zein has shown no melting peak in the DSC thermogram.^42^ Although PVP has a glass transition temperature between 150 and 180°, no endothermic peak was observed in pure PVP between 150 and 200°. PVP has a mild and broad peak between 50 and 100 °C.^19^ DSC thermograms were recorded only for the ZK-TF formulations as these exhibited the most favorable morphology and mechanical integrity.

### FTIR Analysis Result

FTIR analysis of TDF, FTC, polymers, and optimum formulation is shown in Figure 8. The peaks in the FTIR spectrum of TDF were observed to be structurally consistent with the molecular groups. In the FTIR analysis of TDF, Patil and colleagues observed characteristic peaks at 3322, 2985, 1736, 1689, 1267, 1103, 727, 681, and 627 cm^–1^.^43^ The FTIR peaks at 1689 and 1736 cm^–1^ indicate C=O stretching from the fumarate moiety of TDF, the peak at 3322.75 cm^–1^ indicates the NH_2_ stretching vibration, and the peaks at 1267.97 and 1103.88 cm^–1^ are due to C=C stretching in the aromatic ring and C–O group/CH_2_OH stretching, respectively. The peaks around 2985.27, 727.996, and 627.77 cm^–1^ obtained in the study correspond to CH aliphatic stretching, CH_2_ bending, and CH bending, respectively. The peak at 681 cm^–1^ indicates an aromatic ring without plane bending. In the study conducted by Bashir Khan and his colleagues, chitosan nanoparticles were developed for intravaginal application of TDF.^44^ Peaks of TDF were observed at 1373 cm^–1^ (CH3-weak peak), 1170 (P=O stretching), 829 and 979 cm^–1^ (R–CH=CH_2_ bending), and 721 cm^–1^ (C–H bending) in FTIR analyses, similar to our study. Silva et al. reported the absorption bands of TDF in FTIR; it was observed as stretching in the aromatic secondary amine at 3600–3200 cm^–1^, N–H primary amine bending at 1400–1200 and 1650–1580 cm^–1^, C–N bending from the aliphatic tertiary amine at 1250–1020 cm^–1^, and C=O stretching from the ester at 1800–1600 cm^–1^. Peaks compatible with the studies were observed in our study.

FTIR spectrum of FTC shows a broad absorption band for the hydroxyl group between 2710 and 2965 cm^–1^, an NH absorption band at 3280 cm^–1^, an amide (C=O) stretching band in the region of 1640–1690 cm^–1^, a C–F band between 1000 and 1400 cm^–1^, an alkenyl C–H band in the 3010–3100 cm^–1^ region, and an alkenyl C=C stretching band in the range of 1620–1680 cm^–1^.^45^ Similarly, Wang et al. identified characteristic peaks related to the 1,3-oxathiolane ring in the FTIR spectrum of FTC, specifically the C–OH band at 1053 cm^–1^ and the C–O–C band at 1105 cm^–1^.^46^ Fathima et al. reported FTIR peaks for FTC at 1154 cm^–1^ corresponding to C–N stretching, 1629.28 cm^–1^ for C=O stretching, and 3421.27 cm^–1^ for O–H stretching.^41^ Mulik and colleagues identified characteristic signals associated with the structural framework of FTC at 3336 cm^–1^ for O–H, 2111 cm^–1^ for C≡N, and 1637 cm^–1^ for C=C stretching.^47^ The structural characteristic peaks of TDF, FTC, and polymers were observed in FTIR analyses. Peaks consistent with the literature were observed in our study.

Bancila et al. reported that FTIR analysis of zein showed carboxyl groups appeared in the spectrum between 3443 and 3434 cm^–1^ and approximately in the range of 2936 to 2927 cm^–1^. These bands may be influenced by hydrogen bonding in water or interactions with N–H groups. The amide I band was observed between 1633 and 1641 cm^–1^, and bands were identified as a significant feature, primarily associated with C=O stretching vibrations.^48^ The amide II band generally arises from N–H bending and C–N stretching vibrations. Additionally, other important bands related to peptide conformations were found below 1400 cm^–1^. Ali et al. demonstrated in the zein spectrum characteristic bands corresponding to amides I, II, and III at 1643 cm^–1^ (C=O stretching), 1531 cm^–1^ (N–H bending and C–N stretching), and 1240 cm^–1^, respectively.^49^

Kadir et al. showed that the hydroxyl band of PVA was reported at 3354 cm^–1^ in its FTIR spectrum.^50^ Mansur et al. identified the characteristic FTIR bands of PVA as follows: intermolecular and intramolecular hydrogen bonds of O–H groups in the range of 3550–3200 cm^–1^, alkyl group C–H stretching between 2840 and 3000 cm^–1^, C=O stretching bands at 1750–1735 cm^–1^, C–O crystalline bands at 1141 cm^–1^, C–O–C stretching between 1150 and 1085 cm^–1^, and CH_2_ bending vibrations in the range of 1461–1417 cm^–1^.^51^

### Mechanical Characterization of Vaginal Nanofiber Formulations

Mechanical properties (tensile strength and elongation at break) of different zein-based vaginal nanofiber formulations are given as shown in Table 9. While some formulations exhibited measurable and reproducible mechanical properties, others could not be assessed due to their extremely low tensile strength and elongation values, which indicate a brittle structure. The mechanical properties of certain formulations could not be accurately measured due to their extremely low tensile strength and elongation at break, which prevented proper handling during testing. This suggests a highly brittle structure, unlike the other formulations that exhibited measurable and reproducible mechanical values.

**Table 9:** Mechanical Characterization of Zein-Based Vaginal Nanofiber Formulations^a^

formulation code	tensile strength (MPa)	elongation at break (%)
Z1	*	*
Z2	0.501 ± 0.132	0.780 ± 0.439
ZW0	*	*
ZW1	*	*
ZW2	*	*
ZW3	0.691 ± 0.088	2.273 ± 1.239
ZW4	0.644 ± 0.228	0.623 ± 0.031
ZN0	*	*
ZN1	0.989 ± 0.055	43.660 ± 2.983
ZN2	0.650 ± 0.190	4.877 ± 0.206
ZN3	0.8085 ± 0.033	5.583 ± 0.437
ZN4	0.634 ± 0.190	0.210 ± 0.026
ZN5	0.503 ± 0.064	0 ± 0
ZK0	2.866 ± 0.145	17.067 ± 1.508
ZK1	1.471 ± 0.120	3.243 ± 0.585
ZK2	0.302 ± 0.029	0.900 ± 0.329
ZK3	0.607 ± 0.053	2.943 ± 1.639
ZK4	0.396 ± 0.154	0.140 ± 0.242
ZK5	0.682 ± 0.029	0.010 ± 0
ZK6	4.538 ± 0.307	25.823 ± 4.070
ZK7	3.160 ± 0.217	4.950 ± 0.279
ZK8	1.847 ± 0.084	6.417 ± 0.666

a *n* = 3, Mean ± SD, *: Not Detected.

TDF and FTC loading to fibers caused a decreased the mechanical strength of the fiber structure (Table 10). The highest tensile strength was observed in the ZK8-TF formulation (1.525 ± 0.090 MPa) and the lowest value belongs to the ZN3-TF formulation (0.172 ± 0.041 MPa).

**Table 10:** Mechanical Characterization of TDF and FTC-Loaded Zein-Based Vaginal Nanofiber Formulations^a^

formulation code	tensile strenght (MPa)	elongation at break (%)
Z2-TF	0.452 ± 0.110	1.423 ± 2.457
ZN3-TF	0.172 ± 0.041	1.603 ± 0.140
ZK3-TF	0.439 ± 0.059	2.010 ± 0.555
ZK7-TF	1.510 ± 0.501	2.617 ± 0.977
ZK8-TF	1.525 ± 0.090	4.223 ± 2.002

a *n* = 3, Mean ± SD.

### Contact Angles of Vaginal Nanofiber Formulations

Water contact angle values showed surface hydrophilicity of the vaginal nanofiber formulation (Table 11). The ZK4 formulation has the highest contact angle of 100.69°, indicating that the surface is hydrophobic. On the other hand, some formulations of the ZN, ZW, and ZK series are completely wettable.

**Table 11:** Contact Angles of Zein-Based Vaginal Nanofiber Formulations^a^

formulation code	contact angles (°)
Z1	29.15 ± 10.97
Z2	41.39 ± 3.27
ZW0	*
ZW1	0
ZW2	*
ZW3	48.40 ± 1.90
ZW4	63.20 ± 2.53
ZN0	0
ZN1	0
ZN2	63.67 ± 4.73
ZN3	73.19 ± 1.71
ZN4	79.67 ± 4.02
ZN5	51.94 ± 2.24
ZK0	0
ZK1	0
ZK2	49.85 ± 1.09
ZK3	76.76 ± 3.13
ZK4	100.69 ± 3.06
ZK5	20.99 ± 1.94
ZK6	0
ZK7	0
ZK8	0

a *n* = 3, Mean ± SS, *: Not Detected.

Loading the nanofibers with TDF and FTC shows increased surface hydrophilicity, which provides an advantage for accelerated drug release upon contact with water and faster interaction with the mucosal surface (Table 12).

**Table 12:** Contact Angle Values of TDF and FTC-Loaded Zein-Based Vaginal Nanofiber Formulations^a^

formulation code	contact angle (°)
Z2-TF	25 ± 2.23
ZN3-TF	0
ZK3-TF	0
ZK7-TF	0
ZK8-TF	0

a *n* = 3, Mean ± SD, *: Not Detected.

### In Vitro Release Studies of Formulation

The in vitro release profiles of TDF- and FTC-loaded zein-based vaginal nanofiber formulations demonstrated sustained and controlled drug release over 24 h, a critical feature for effective vaginal pre-exposure prophylaxis (PrEP) against HIV (Figure 9). The release varied depending on polymer composition, fiber diameter, and drug–polymer interactions, indicating that these nanofiber systems can be tailored for vaginal drug delivery applications.

### Mucoadhesion Studies of Formulations

Mucoadhesion studies were performed exclusively on the TDF- and FTC-loaded nanofiber formulations, as these exhibited the most favorable morphological and mechanical characteristics, indicating the highest potential for vaginal application. (Figure 10). This selection was based on key properties that influence mucoadhesion, such as regular fiber morphology, appropriate fiber diameter, high porosity, and enhanced mechanical strength. Our study focused on systems that provide effective local drug release and prolonged mucosal contact time for the prevention of sexually transmitted HIV infection.

## Discussion

The successful fabrication of uniform zein-based nanofibers via electrospinning is closely influenced by the physicochemical characteristics of polymer solutions, including viscosity, conductivity, and surface tension. Conductivity indicates the charge density and repulsion of charges on the surface of the jet during the electrospinning process.^52^ The polymer solution is stretched by the repulsion of surface charges, and a higher solution conductivity allows for greater charge-carrying capacity. Studies have shown that fibers with smaller diameters and beadless form can be produced by increasing the conductivity of the solution.^53^ Conductivity is dependent on the ionic character of the solution and the interactions between the polymers. In the hybrid nanofiber formulations with zein, while the PEO and PVP concentrations were constant, an increase in conductivity values was observed due to the increase in zein concentration. A decrease in the conductivity of the solutions was observed with the addition of PVP and PEO at different rates relative to the polymer solutions prepared with the same zein concentration. PEO exhibits low conductivity values due to its nonionic nature. The decrease in solution conductivity with the increasing PVP concentration was associated with the nonconductive nature of the polymer. In electrospinning, insufficient viscosity of the polymer solution may lead to discontinuous fiber formation, whereas high viscosity prevents the flow of the polymer. The viscosity must be sufficiently high to overcome the surface tension by a synergistic effect with electrostatic repulsion.^54^ The viscosity of zein-based hybrid nanofiber formulations increased with the addition of PEO and PVP, which were added to enhance the mechanical strength and mucoadhesive properties. Rheological characterization of zein solutions further demonstrated a concentration-dependent behavior, with higher zein concentrations resulting in elevated viscosity values.^55^ During the electrospinning process, the surface tension must be overcome by the electric field forces, which subsequently influence the properties of nanofibers.^56^ Zein-based hybrid nanofiber formulations were prepared with the use of a solvent system composed of a water–ethanol mixture. Since the solvent system remained the same for all zein-based formulations, the surface tension values of the polymer solutions were found to be very similar. According to Jaworek and colleagues, the surface tension values for zein formulations were suitable for electrospinning, as liquids with surface tension values higher than 50 mN/m cannot be atomized by electrical forces.^57^ They attributed this to the presence of ethanol, which has a lower surface tension compared with distilled water. It was observed that the addition of TDF and FTC to the polymer solutions led to an increase in the surface tension. A higher surface tension necessitates a stronger electric field for successful electrospinning. TDF and FTC loading the fibers altered the solution viscosity, surface tension, and conductivity. Tuğcu Demiröz and colleagues developed metronidazole-loaded vaginal nanofiber formulations. An increase in conductivity values was observed by loading metronidazole into the polymer solution.^19^ In our study, the highest increase in surface tension values was observed with the addition of TDF and FTC. Since the same solvent system was used to prepare the polymer solution, the surface tension values were found to be similar in all formulations. Birer and Acartürk developed a telmisartan loaded polycaprolactone/gelatin based electrospun vascular scaffold.^58^ Similarly, in their study, surface tension values were found to be similar, depending on the same solvent system used in our formulation.

In our study, zein-PEO (MW:600 kDa) nanofiber showed ribbon-like structures (Figure 3). The nanofibers exhibited a highly uniform morphology, characterized by relatively small and evenly distributed diameters. Bead formation was not observed in the SEM images, indicating a good optimization of the electrospinning parameters. The average diameter of fibers fell predominantly within the range of 400–600 nm. This observation suggests a high degree of homogeneity in the fiber dimensions, which is consistent with controlled electrospinning. Yao et al. developed zein nanofibers via electrospinning using ethanolic aqueous solutions with ethanol/water ratios of 80:20 and 90:10 (v/v) and zein concentrations of 20, 30, and 40% (w/v).^59^ They reported that increasing the zein concentration above 30% resulted in ribbon-like nanofibers with diameters ranging from approximately 1 to 6 μm and noted that fiber morphology was influenced by solution concentration. The resulting zein nanofibers were observed to be glossy in appearance. Despite the varying solvent compositions, the nanofiber morphologies were found to be similar. Similarly, in our formulations containing a high zein content, thick and ribbon-shaped fibers were observed, characterized by reduced porosity and increased diameter. These results confirm that fiber morphology is strongly influenced by zein concentration and that higher polymer content promotes the formation of flattened, ribbon-like structures due to increased viscosity and reduced jet stretching during electrospinning. The images of nanofibers show homogeneous fiber formation with minimal bead defects and narrow diameter distributions in zein- PEO nanofibers, as seen in Figure 4. The fibers are quite thin and homogeneous. Fiber diameter distribution for the ZN2 formulation is in the range of 200–600 nm. Regular and thin fibers are formed thanks to the stable solution obtained with low zein and PEO concentration. This fiber structure can offer the advantage of a faster release due to larger surface area. For the ZN4 formulation, the fibers are regular, with slight diameter differences observed. In the ZN5 formulation, the fibers are significantly thickened, and ribbon-like structures are observed. In this formulation with the highest zein content, the high viscosity and low conductivity of the solution limited the elongation of the fibers, resulting in very thick and less porous fibers. This structure may be advantageous in terms of long-term release and the mucosal barrier effect. Broken fiber structures indicating the low mechanical properties of zein nanofibers are observed in SEM images as seen in Figure 5. The diameter of the fibers varies in the range of 300–700 nm, and a narrow distribution is seen. ZK1 presents the most homogeneous and finely structured nanofibers. ZK4 thickened significantly and transitioned to a ribbon-like structure. The fibers are regular, ribbon-shaped, and have shiny surfaces. ZK4 shows ribbon-like structures due to the increased zein ratio. In ZK5, the fibers are both thicker and more irregular. Especially in image A, the fibers are seen to be twisted with beads and coalescence in some places. SEM images confirmed the coexistence of ribbon-like and round fiber morphologies among the zein-based formulations, consistent with previously reported zein electrospun systems.^60–62^ Wang et al. investigated the modified coaxial electrospinning of zein using zein solution as the core fluid and LiCl solutions as the sheath fluids. They demonstrated that the width of the resulting zein nanoribbons was strongly dependent on the sheath fluid conductivity and LiCl concentration, showing clear linear correlations with both parameters. Moreover, nanoribbon dimensions were closely associated with process characteristics such as spreading angle and jet length, highlighting the critical role of electrohydrodynamic conditions in determining ribbon morphology.^60^ For the ZK7 formulation, the fiber diameter is in the range of 300–800 nm, and the fiber distribution is narrow. ZK2-TF, fiber diameter is generally between 400 and 800 nm, and the distribution is quite narrow (Table 8). Homogeneity in TDF and FTC-loaded formulations provides an advantage for the regular release of the drug. ZN3-TF, fibers are slightly thicker and irregularly arranged (Figure 6). Fiber diameters are approximately 500–1100 nm. In ZK3-TF, fibers are medium-thick, regularly arranged, and no beads were seen in the SEM images. Fiber orientation is random, but the structural integrity is preserved. The ZK8-TF fibers are morphologically quite regular but have a large diameter. This may slow the release of TDF and FTC from fibers and provide a more permanent structure during mucosal application. The high viscosity and high conductivity contributed to a smooth and bead-free fiber structure. In the formulations prepared with zein and PEO, the low conductivity of PEO reduced the total conductivity of the solution and caused the formation of thicker fibers. The increase in the fiber diameter can be explained together with viscosity and surface tension. The high viscosity of the fibers prepared with zein and PVP caused the formation of thick fibers. The high surface tension required a stronger electric field for electrospinning.

**Figure 3:**
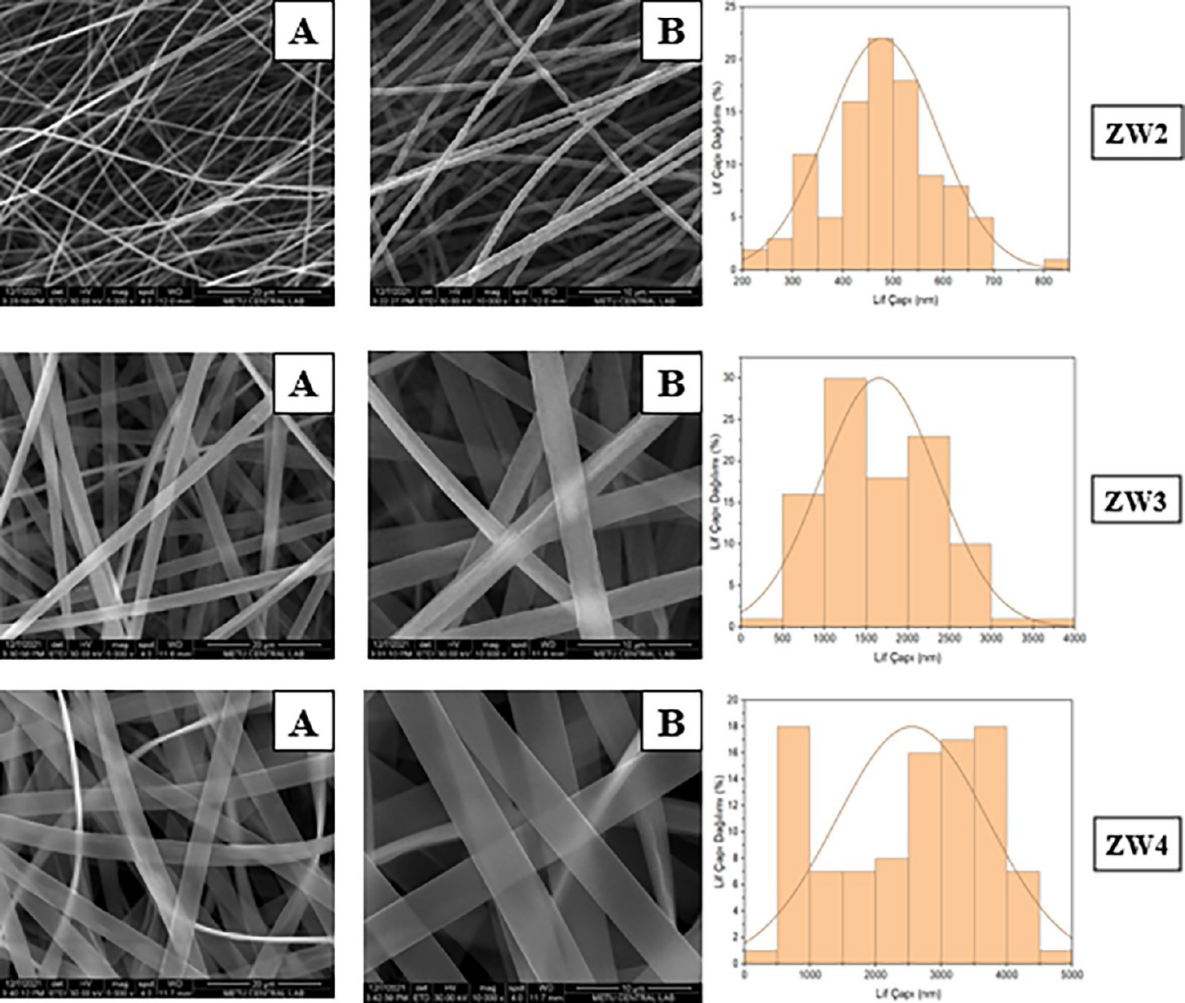
SEM images and fiber diameter distributions of Zein-PEO (ZW series) (MW:600 kDa) based nanofibers (A: 5000×, B: 10,000×).

**Figure 4:**
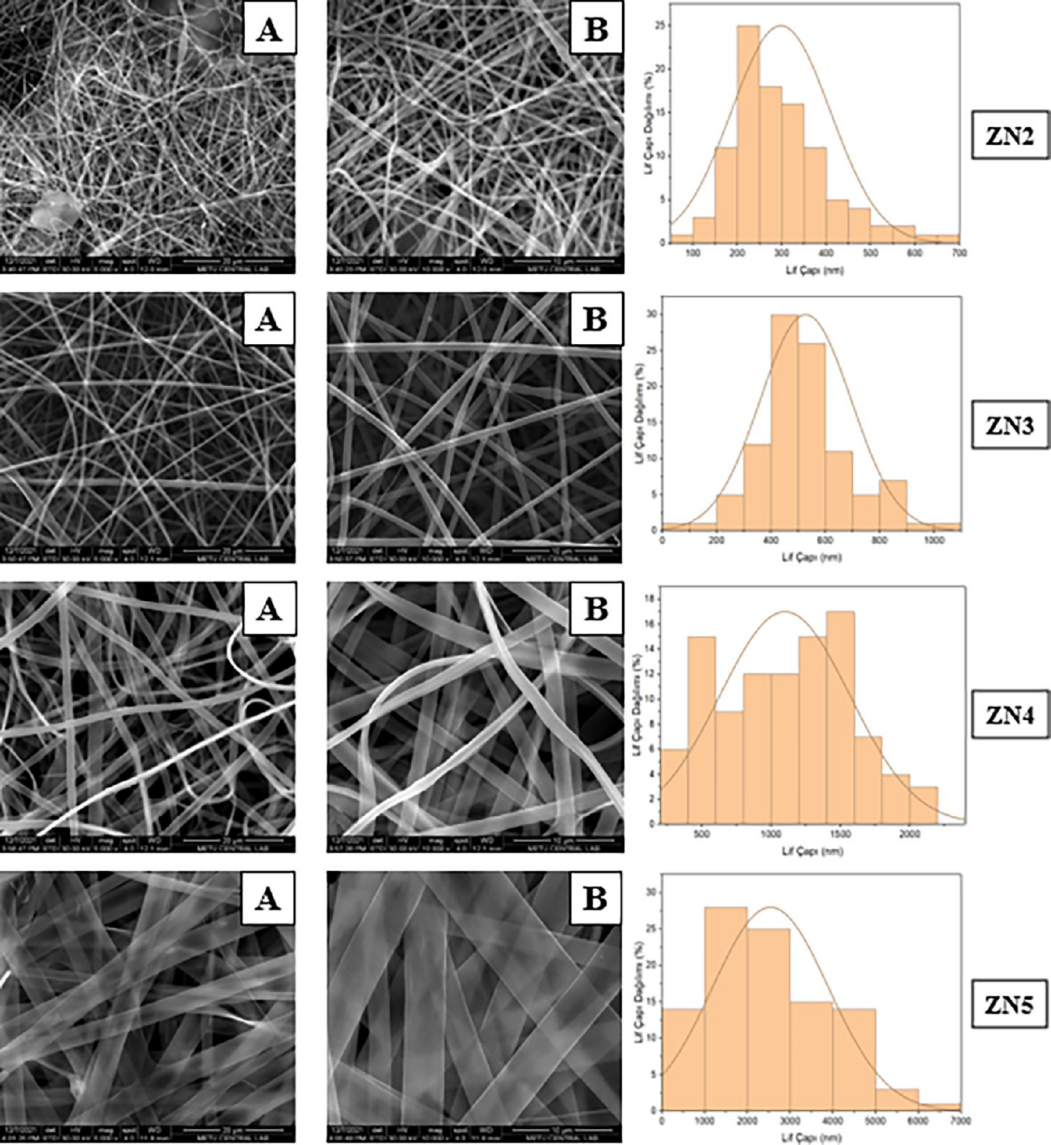
SEM images and fiber diameter distributions of zein-PEO (ZN series) (MW:200 kDa) based nanofibers (A: 5000×, B: 10,000×).

**Figure 5:**
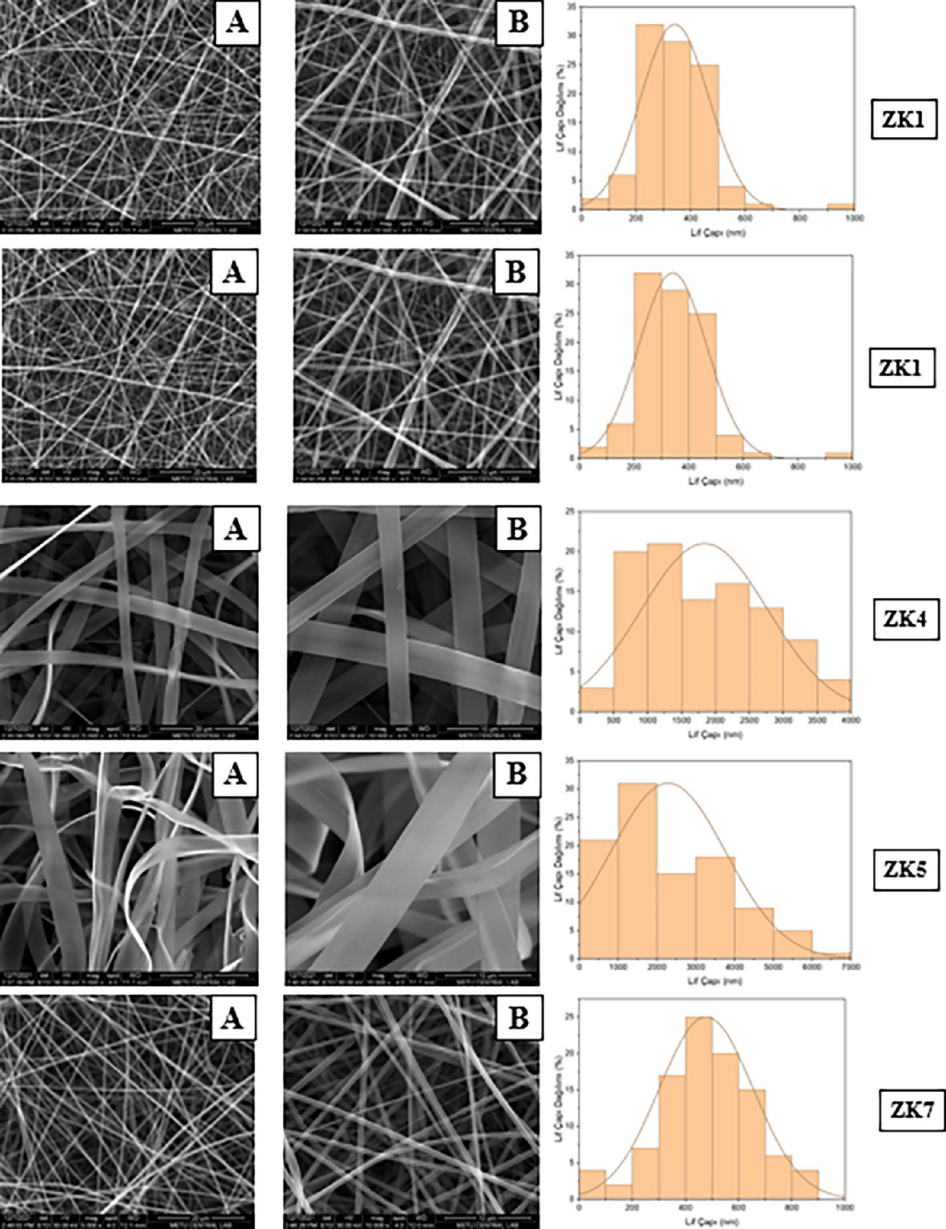
SEM images and fiber diameter distributions of zein-PVP (ZK series) based nanofibers (A: 5000×, B: 10,000×).

**Figure 6:**
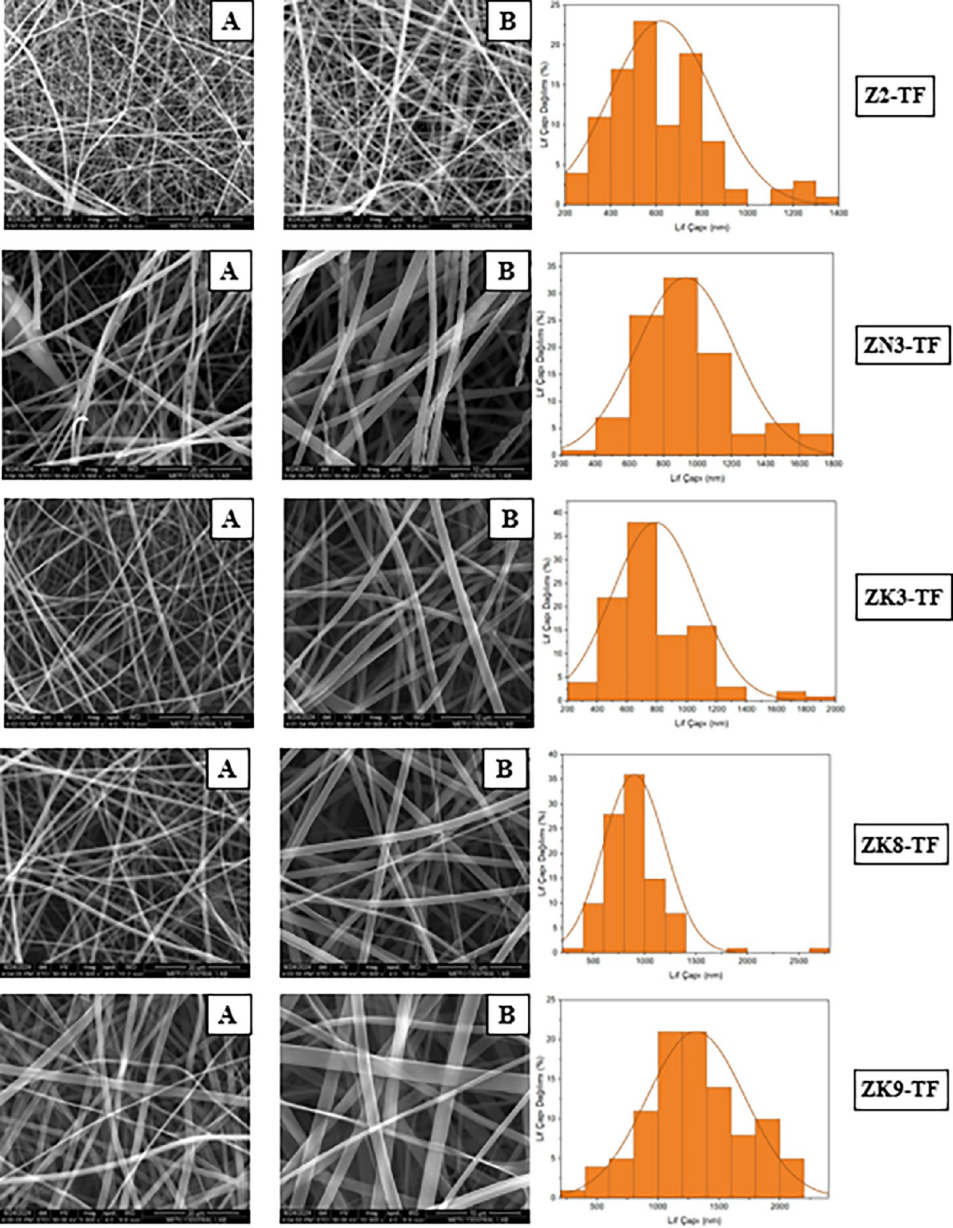
SEM images and fiber diameter distributions of TDF and FTC-loaded zein-based nanofibers (A: 5000×, B: 10,000×).

The porosity values of polymeric nanofibers are approximately 50–60%.^63^ The porosity measurements of the nanofibers we prepared vary between 40 and 75% depending on the type of polymer. The porosity values of the vaginal nanofiber formulations were suitable for vaginal application, ensuring sufficient fluid penetration and an effective mucoadhesive interaction. With the addition of TDF and FTC, the surface tension of the solution also increased, and this situation caused the need for a stronger electrostatic force and affected the fiber morphology. Compared with blank formulations with similar compositions, the TDF- and FTC-loaded formulations exhibited increased fiber diameters. For instance, ZK3 (blank) had an average fiber diameter of 581.68 ± 268.43 nm, while ZK3-TF increased to 790.95 ± 289.82 nm following drug loading into the formulation. Huang et al. produced pH-sensitive cellulose acetate phthalate nanofiber containing TDF; they did not show any change in fiber diameter compared to blank fibers.^64^ Fiber diameter has a critical role in the release properties of active substances, and decreasing fiber diameter increases the release of active substances.^65^ This is due to an increase in surface tension and viscosity in the polymer solutions with drug addition, which in turn inhibits full jet stretching and favors thicker fiber formation (Figures 7–10).

**Figure 7:**
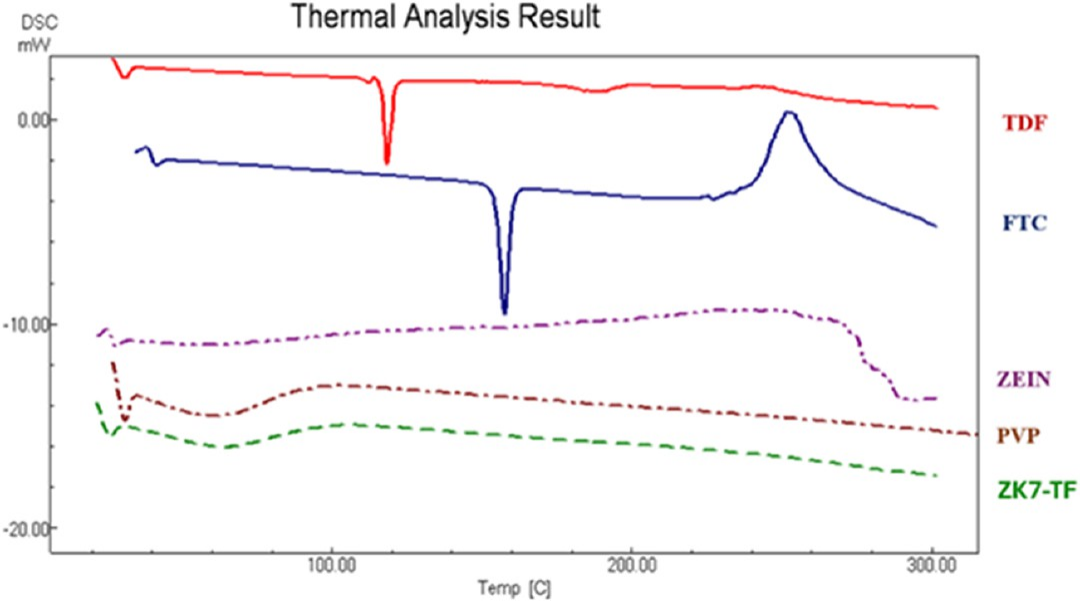
DSC thermogram of TDF, FTC, Zein, PVP, and ZK7-TF vaginal nanofiber formulations.

**Figure 8:**
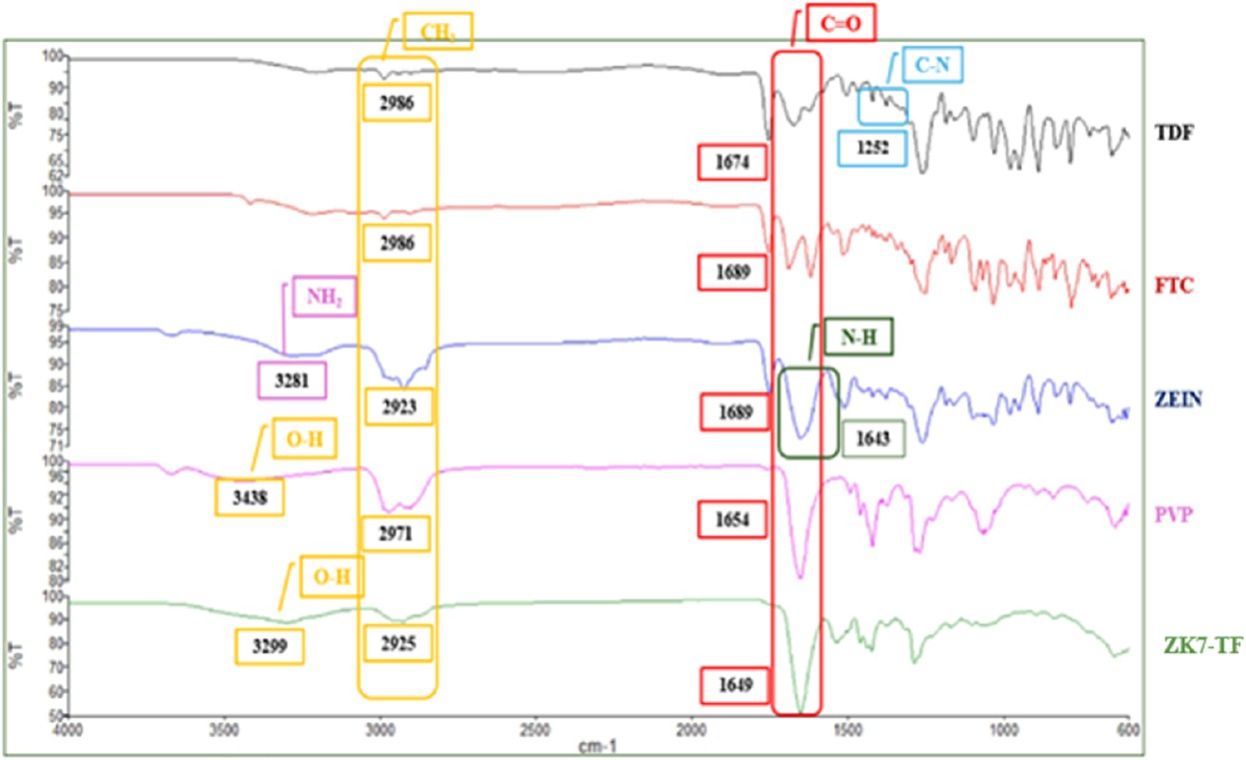
FTIR spectrum of TDF, FTC, Zein, and ZK7-TF vaginal nanofiber formulations.

**Figure 9:**
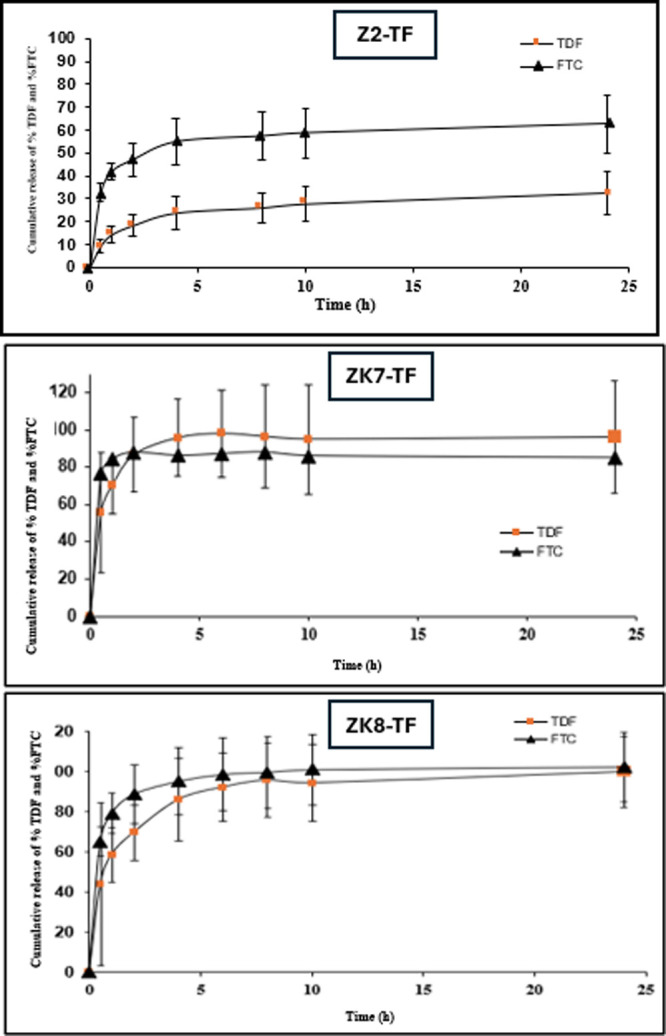
TDF and FTC release profiles from zein-based vaginal nanofiber formulation (*n* = 3, Mean ± SD).

**Figure 10:**
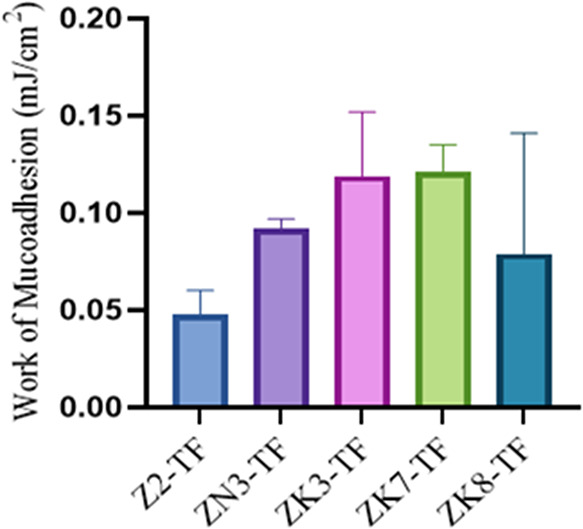
Work of mucoadhesion of TDF and FTC-loaded vaginal nanofiber formulation using cow vaginal tissue (*n* = 3, Mean ± SD).

Sharp endothermic peaks specific to TDF and FTC formulations were not observed in the DSC thermogram. As zein and PVP are amorphous polymers, they do not exhibit sharp crystalline melting transitions in DSC thermograms, and therefore, their degree of crystallinity cannot be calculated from DSC. The disappearance of melting peaks of TDF and FTC indicates that these drugs were integrated into the formulation in an amorphous or molecularly dispersed form and interacted with the polymer matrix. In the FTIR spectrum of the formulations, the broad O–H stretch band (3299 cm^–1^) indicates the presence of hydroxyl groups from zein and PVP polymers and drugs. This band shows a slight shift in the K7-TF spectrum. This may be an indication of hydrogen bonds or intermolecular interactions. In the FTIR spectrum of the ZK7-TF formulation, the disappearance or shift of the characteristic peaks corresponding to TDF (1674 cm^–1^) and FTC (1689 and 1252 cm^–1^) suggests a successful loading of the drugs into the polymeric matrix.

Tensile strength of formulations is generally in the range of 0.3–1.5 MPa. Studies have shown that plant-derived proteins such as zein generally suffer from poor mechanical properties, including low tensile strength and limited elongation at break, which significantly restrict their structural stability.^66–68^ By adding hydrophilic copolymers such as PVP and PEO to zein-structured nanofiber formulations, intermolecular interactions between fibers are strengthened, thus increasing the flexibility, tensile strength, and integrity of the fibers. Voght et al. developed electrospun zein fibers incorporating poly­(glycerol sebacate) for soft tissue engineering.^69^ Tensile testing of neat zein revealed a Young’s Modulus of 22 ± 7 MPa, an ultimate tensile strength of 0.3 ± 0.1 MPa, and a failure strain of 2.7 ± 0.9%. They reported much lower values for the mechanical properties. The ZK series prepared with PVP and zein exhibits better performance, especially in terms of tensile strength and elongation at break. This shows that the structural integrity and elasticity of the ZK formulations are superior to those of the other formulations. The ZN1 formulation stands out with its high elasticity, which shows that it is a more flexible and extensible fiber formulation. These differences show that the polymer ratios in the formulation affect the mechanical strength. Drug loading generally reduces the elongation at the break value. This may be due to restricted mobility within the polymer matrix, increased crystallinity, or structural weakening of the drugs. ZK8-TF stands out because it has both the highest tensile strength and elongation, while ZN3-TF is a formulation that exhibits low mechanical properties with low strength and low elongation. PVP-zein formulation series showed much higher mechanical strength than the other formulations. This is probably related to changes in the polymer and zein ratios used in the formulation. Formulations that have low tensile strength and elongation at break values are likely to exhibit brittle behavior during application. Tuğcu-Demiröz et al. developed benzydamine-loaded vaginal nanofiber formulations.^70^ The tensile strength of these nanofibers was between 1.749 and 2.406 MPa, while their elongation at break was between 28 and 39%. Loading the formulation with benzydamine in the form of nanoparticles resulted in a lower tensile strength and elongation at break.

The increase in the contact angle in the ZN and ZK series, depending on the formulation content, shows that the formulation composition directly affects the surface properties. Zein-based nanofibers exhibit high solubility or dispersibility in water, indicating potential advantages for drug delivery applications. Ali et al. reported an initial contact angle of 115° in their study on zein nanofibers.^49^ However, the contact angle of the zein-containing nanofibers dropped to nearly zero within one second. This change in the contact angle is attributed to the poor morphological stability of zein in water. Incorporation of hydrophilic drugs or polymers into electrospun fibers enhances the surface wettability in mucosal applications. In the study conducted by Dragar et al., the incorporation of hydrophilic drugs, paracetamol and metformin, into nanofibers resulted in a reduced contact angle compared to the blank formulation, indicating increased surface hydrophilicity.^71^ In our study, loading of hydrophilic drugs into nanofibers increased surface wettability, which provided advantages such as rapid contact with the vaginal mucosa. Increased surface hydrophilicity generally facilitates faster water penetration and drug dissolution, leading to an accelerated initial release; however, excessive wettability may compromise prolonged mucoadhesion by reducing the residence time on the mucosal surface.

Drug release profiles were influenced by both the polymer composition and fiber morphology. Zein-PVP-based formulations showed slower release rates compared to zein-only or zein-PEO systems, attributed to stronger drug-polymer interactions with PVP. Zein-PEO formulations exhibited faster release, likely due to the hydrophilic nature of PEO, which enhances water uptake and drug diffusion. Smaller fiber diameters (Z2-TF: 622 nm) correlated with faster drug release due to higher surface area-to-volume ratios. Higher fiber diameters (ZK9-TF: 1312 nm) and ribbon-like structures caused delayed release as thicker fibers reduce release rates and pore accessibility. These findings are consistent with previous reports, highlighting that nanofiber wettability, porosity, and diameter determine burst and sustained release.^72^ The ZK7-TF formulation also demonstrated a desirable sustained-release profile with controlled drug diffusion over 24 h. Its fiber diameter and uniform morphology enabled a balance between initial hydration and drug retention, supporting once-daily local PrEP application. A positive relationship was observed between mucoadhesive strength and controlled drug release, as the ZK7-TF formulation with higher adhesion values also exhibited a more controlled release profile compared to ZK8-TF. This can be attributed to the intimate contact of the nanofiber matrix with the mucosal surface, which prolongs the residence time and enhances local drug availability, consistent with previous reports on mucoadhesive nanofibrous systems. By contrast, Z2-TF showed the lowest mucoadhesion but demonstrated the most sustained release behavior, likely due to its polymer composition. These findings indicate that while higher mucoadhesion often correlates with enhanced local availability, the hydrophilicity of the polymer matrix also plays a critical role in achieving controlled and effective drug release.^73,74^

Mucoadhesion refers to the adhesive strength of a material to mucosal surfaces; a higher value indicates stronger and more durable adhesion. High mucoadhesion provides advantages in terms of increasing the local effect of the drug and the system remaining in the mucosa for a longer time. ZK3-TF and ZK7-TF formulations have the highest mucoadhesion works, 0.119 and 0.121 mJ/cm^2^, respectively. The lowest mucoadhesion was observed in the ZK8-TF formulation (0.079 mJ/cm^2^). In the PEO-based ZN3-TF formulation, the mucoadhesion value was determined as 0.092 mJ/cm^2^. Especially, ZK7-TF shows superiority in terms of both mechanics and mucoadhesion. Among all TDF and FTC-loaded nanofiber formulations, ZK7-TF exhibited the highest mucoadhesion performance, demonstrating a superior ability to establish prolonged contact with the vaginal mucosa. ANOVA analysis of mucoadhesion values indicated that the differences among formulations were not statistically significant (*p* > 0.05). Although variations in mean adhesion values were observed, these did not reach statistical significance, which may be attributed to experimental variability and the relatively close performance of the different polymer compositions. This enhanced mucoadhesion can be attributed to the composition of zein and PVP, which provides sufficient mechanical integrity while maintaining surface wettability to support mucosal interactions. Tort and Acartürk developed chitosan-containing glutamine-loaded nanofibers that showed low mucoadhesion and mechanical properties. It was thought that these nanofibers showed poorer mechanical properties compared to other formulations due to the lowest average diameter.^25^ Z2-TF is the formulation with the smallest fiber diameter (622 nm) and the lowest workability of mucoadhesion. When the fiber diameter becomes excessively small, the interfiber network structure may weaken, which could reduce mechanical strength and mucoadhesion to the mucosal surface.

## Conclusions

The novelty of this work is the use of zein, a plant-derived biopolymer primarily studied for oral and transdermal systems, as the main matrix for vaginal mucoadhesive nanofibers for PrEP. The poor mechanical properties and brittle nature of zein have been successfully overcome by incorporating hydrophilic polymers such as PEO and PVP, which improve fiber flexibility, tensile strength, and overall structural integrity without compromising mucoadhesion or controlled drug release properties. In this respect, the study not only offers a novel drug delivery platform but also demonstrates that zein can be optimized for vaginal application. This highlights a new direction in the application of zein for vaginal PrEP formulations.

This study was based on the hypothesis that incorporating TDF and FTC into zein-based electrospun nanofibers, reinforced with mucoadhesive polymers, would provide a suitable platform for vaginal PrEP. We further hypothesized that the combination of strong mucoadhesion and controlled release behavior would ensure prolonged local drug retention and enhance prophylactic efficacy.

Our findings confirmed the hypothesis that coloading TDF and FTC into zein-based nanofibers reinforced with mucoadhesive polymers provides a feasible platform for vaginal PrEP. These outcomes not only align with prior reports on mucoadhesive electrospun systems but also validate zein’s potential as a versatile polymer for vaginal drug delivery.

Future studies should prioritize in vivo pharmacokinetic and pharmacodynamic evaluations, along with scale-up of production processes, to translate this platform toward clinical application. These findings suggest that zein-based hybrid nanofibers represent a safe, effective, and patient-compliant strategy for enhancing adherence and efficacy in HIV pre-exposure prophylaxis.

## Abbreviations

AIDS: acquired immunodeficiency syndrome
TDF: tenofovir disoproxil fumarate
FTC: emtricitabine
HIV: human immunodeficiency virus
PrEP: pre-exposure prophylaxis
PVP: polyvinylpyrrolidone, FTIR, Fourier transform infrared spectroscopy
DSC: differential scanning calorimetry
SEM: scanning electron microscopy, PEO, poly­(ethylene oxide)
SVF: simulated vaginal fluid
HPLC: high-performance liquid chromatography

## References

[1] Kumari G., Singh R. K. (2012). Highly active antiretroviral therapy for treatment
of HIV/AIDS patients: current status and future prospects and the
Indian scenario. HIV AIDS Rev..

[2] Jones H. S., Anderson R. L., Cust H., McClelland R. S., Richardson B. A., Thirumurthy H., Malama K., Hensen B., Platt L., Rice B. (2024). HIV incidence among
women engaging in sex work in sub-Saharan Africa: a systematic review
and meta-analysis. Lancet Glob. Health.

[3] Smith A. D., Tapsoba P., Peshu N., Sanders E. J., Jaffe H. W. (2009). Men who
have sex with men and HIV/AIDS in sub-Saharan Africa. Lancet.

[4] Cautela M. P., Moshe H., Sosnik A., Sarmento B., Das Neves J. (2019). Composite
films for vaginal delivery of tenofovir disoproxil fumarate and emtricitabine. Eur. J. Pharm. Biopharm..

[5] Adams J. L., Shelley K., Nicol M. R. (2019). Review of Real-World
Implementation
Data on Emtricitabine-Tenofovir Disoproxil Fumarate as HIV Pre-exposure
Prophylaxis in the United States. Pharmacotherapy.

[6] Nunes R., Bogas S., Faria M. J., Gonçalves H., Lúcio M., Viseu T., Sarmento B., Das Neves J. (2021). Electrospun
fibers for vaginal administration of tenofovir disoproxil fumarate
and emtricitabine in the context of topical pre-exposure prophylaxis. J. Controlled Release.

[7] Khan A. B., Thakur R. S. (2014). Formulation and evaluation of mucoadhesive
vaginal
tablets of tenofovir disoproxil fumarate. Der
Pharm. Lett..

[8] Baum M. M., Butkyavichene I., Churchman S. A., Lopez G., Miller C. S., Smith T. J., Moss J. A. (2015). An intravaginal ring for the sustained
delivery of tenofovir disoproxil fumarate. Int.
J. Pharm..

[9] Avlani D., Kumar A., HN S. (2023). Development of dispersible
vaginal
tablets of tenofovir loaded mucoadhesive chitosan microparticles for
anti-HIV pre-exposure prophylaxis. Mol. Pharmaceutics.

[10] Okenwa L., Lawoko S., Jansson B. (2011). Contraception,
reproductive health
and pregnancy outcomes among women exposed to intimate partner violence
in Nigeria. Eur. J. Contracept. Reprod. Health
Care.

[11] Celum C., Baeten J. M. (2012). Tenofovir-based pre-exposure prophylaxis
for HIV prevention:
evolving evidence. Curr. Opin Infect Dis.

[12] Clark M. R., Peet M. M., Davis S., Doncel G. F., Friend D. R. (2014). Evaluation
of rapidly disintegrating vaginal tablets of tenofovir, emtricitabine
and their combination for HIV-1 prevention. Pharmaceutics.

[13] Huang Z.-M., Zhang Y.-Z., Kotaki M., Ramakrishna S. (2003). A review on
polymer nanofibers by electrospinning and their applications in nanocomposites. Compos. Sci. Technol..

[14] Venmathi
Maran B. A., Jeyachandran S., Kimura M. (2024). A review on the electrospinning
of polymer nanofibers and its biomedical applications. J. Compos Sci..

[15] Xue J., Wu T., Dai Y., Xia Y. (2019). Electrospinning and electrospun nanofibers:
Methods, materials, and applications. Chem.
Rev..

[16] Tort S., Acartürk F., Beşikci A. (2017). Evaluation
of three-layered doxycycline-collagen
loaded nanofiber wound dressing. Int. J. Pharm..

[17] Razavi M. S., Abdollahi A., Malek-Khatabi A., Ejarestaghi N. M., Atashi A., Yousefi N., Ebrahimnejad P., Elsawy M. A., Dinarvand R. (2023). Recent advances in PLGA-based nanofibers
as anticancer drug delivery systems. J. Drug
Delivery Sci. Technol..

[18] Sumathi S. (2025). A Review of
Electrospun Polymeric Fibers as Potential Drug Delivery Systems for
Tunable Release Kinetics. J. Sci.:Adv. Mater.
Devices.

[19] Tuğcu-Demiröz F., Saar S., Tort S., Acartürk F. (2020). Electrospun
metronidazole-loaded nanofibers for vaginal drug delivery. Drug Dev. Ind. Pharm..

[20] Dong Y., Jaleh B., Ashrafi G., Kashfi M., Rhee K. Y. (2025). Mechanical
properties of the hybrids of natural (alginate, collagen, chitin,
cellulose, gelatin, chitosan, silk, and keratin) and synthetic electrospun
nanofibers: A review. Int. J. Biol. Macromol..

[21] Azizi H., Koocheki A., Ghorani B. (2023). Structural
elucidation of Gluten/Zein
nanofibers prepared by electrospinning process: Focus on the effect
of zein on properties of nanofibers. Polym.
Test.

[22] Bhatnagar S., Kumari P., Pattarabhiran S. P., Venuganti V. V. K. (2018). Zein
microneedles for localized delivery of chemotherapeutic agents to
treat breast cancer: drug loading, release behavior, and skin permeation
studies. AAPS PharmSciTech..

[23] Esposito D., Conte C., d’Angelo I., Miro A., Ungaro F., Quaglia F. (2020). Mucoadhesive zein/beta-cyclodextrin
nanoparticles for
the buccal delivery of curcumin. Int. J. Pharm..

[24] Falsafi S. R., Topuz F., Esfandiari Z., Can Karaca A., Jafari S. M., Rostamabadi H. (2023). Recent trends
in the application
of protein electrospun fibers for loading food bioactive compounds. Food Chem.:X.

[25] Tort S., Acartürk F. (2016). Preparation and characterization
of electrospun nanofibers
containing glutamine. Carbohydr. Polym..

[26] Lin S.-H., Ou S.-L., Hsu H.-M., Wu J.-Y. (2023). Preparation and
characteristics of polyethylene oxide/Curdlan nanofiber films by electrospinning
for biomedical applications. Materials.

[27] Nainggolan G., Gea S., Marpongahtun, Harahap M., Dellyansyah, Situmorang S. A. (2023). Promoting electrospun lignin/PEO
nanofiber for high-performance CO filtration. J. Nat. Fibers.

[28] Tuğcu-Demiröz F. (2017). Vaginal delivery
of benzydamine hydrochloride through liposomes dispersed in mucoadhesive
gels. Chem. Pharm. Bull..

[29] Eskitoros-Togay Ş.
M., Bulbul Y. E., Tort S., Korkmaz F. D., Acartürk F., Dilsiz N. (2019). Fabrication of doxycycline-loaded electrospun PCL/PEO
membranes for a potential drug delivery system. Int. J. Pharm..

[30] Virginia C., Khasanah A., Jauhari J., Sriyanti I. (2020). Electrospinning and
characterization nanofibers and nano particle of Polyvinylpyrrolidone. IOP Conf. Ser.: Mater. Sci. Eng..

[31] Destache C. J., Mandal S., Yuan Z., Kang G., Date A. A., Lu W., Shibata A., Pham R., Bruck P., Rezich M. (2016). Topical
tenofovir disoproxil fumarate nanoparticles prevent HIV-1
vaginal transmission in a humanized mouse model. Antimicrob. Agents Chemother..

[32] Kanjanapongkul K., Wongsasulak S., Yoovidhya T. (2010). Investigation and prevention of clogging
during electrospinning of zein solution. J.
Appl. Polym. Sci..

[33] Tort S., Yıldız A., Tuğcu-Demiröz F., Akca G., Kuzukıran Ö., Acartürk F. (2019). Development
and characterization of rapid dissolving ornidazole loaded PVP electrospun
fibers. Pharm. Dev. Technol..

[34] Gajewski A. (2017). A couple new
ways of surface tension determination. Int.
J. Heat Mass Transfer.

[35] Salam A., Khan M. Q., Hassan T., Hassan N., Nazir A., Hussain T., Azeem M., Kim I. S. (2020). In-vitro assessment
of appropriate hydrophilic scaffolds by co-electrospinning of poly­(1,4
cyclohexane isosorbide terephthalate)/polyvinyl alcohol. Sci. Rep..

[36] Tuğcu-Demiröz F., Acartürk F., Erdoğan D. (2013). Development of long-acting bioadhesive
vaginal gels of oxybutynin: Formulation, in vitro and in vivo evaluations. Int. J. Pharm..

[37] Cevher E., Sensoy D., Taha M. A., Araman A. (2008). Effect of thiolated
polymers to textural and mucoadhesive properties of vaginal gel formulations
prepared with polycarbophil and chitosan. AAPS
PharmSciTech..

[38] Rüzgar
Özemre G., Kara A. A., Pezik E., Tort S., Vural İ., Acartürk F. (2023). Preparation of nanodelivery systems
for oral administration of low molecular weight heparin. J. Drug Delivery Sci. Technol..

[39] Lee E. H., Smith D. T., Fanwick P. E., Byrn S. R. (2010). Characterization
and anisotropic lattice expansion/contraction of polymorphs of tenofovir
disoproxil fumarate. Crystal Growth Des..

[40] Silva J. P.
A., Figueirêdo C. B. M., De Medeiros Vieira A. C. Q., De Lyra M. A. M., Rolim L. A., Rolim-Neto P. J., De La Roca Soares M.
F., Albuquerque M. M., Soares-Sobrinho J. L. (2017). Thermal
characterization and kinetic study of the antiretroviral tenofovir
disoproxil fumarate. J. Therm. Anal. Calorim..

[41] Fathima A., Hari B. V., Devi D. R. (2014). Development of microparticulate sustained
release dosage form of emtricitabine: An anti-HIV drug. Asian J. Chem..

[42] Hu D., Lin C., Liu L., Li S., Zhao Y. (2012). Preparation,
characterization,
and in vitro release investigation of lutein/zein nanoparticles via
solution enhanced dispersion by supercritical fluids. J. Food Eng..

[43] Patil S., Kadam C., Pokharkar V. (2017). QbD based
approach for optimization
of Tenofovir disoproxil fumarate loaded liquid crystal precursor with
improved permeability. J. Adv. Res..

[44] Khan A., Sharnagat Thakur R. (2014). Formulation
and evaluation of mucoadhesive microspheres
of tenofovir disoproxil fumarate for intravaginal use. Curr. Drug Deliv.

[45] Al-Majed A. A., Bakheit A. H., Al-Qahtani B. M., Al-Kahtani H. M., Abdelhameed A. S. (2020). Chapter Three - Emtricitabine. Profiles Drug Subst., Excipients, Relat. Methodol..

[46] Wang X.-J., You J.-z., Yu F. (2015). Study on the thermal decomposition
of emtricitabine. J. Anal. Appl. Pyrolysis.

[47] Mulik B. B., Dhumal S. T., Harale R. R., Kharat K. R., Sathe B. R. (2018). Electrochemical
Studies of Anti-HIV Drug Emtricitabine: Oxidative Determination and
Improved Antimicrobial Activity. ChemElectroChem..

[48] Bancila S., Ciobanu C.-I., Murariu M., Drochioiu G. (2016). Ultrasound-assisted
zein extraction and determination in some patented maize flours. Rev. Roum Chim.

[49] Ali S., Khatri Z., Oh K. W., Kim I.-S., Kim S. H. (2014). Zein/cellulose
acetate hybrid nanofibers: Electrospinning and characterization. Macromol. Res..

[50] Kadir M. F. Z., Aspanut Z., Majid S. R., Arof A. K. (2011). FTIR studies
of
plasticized poly­(vinyl alcohol)–chitosan blend doped with NH4NO3
polymer electrolyte membrane. Spectrochim. Acta,
Part A.

[51] Mansur H. S., Sadahira C. M., Souza A. N., Mansur A. A. (2008). FTIR spectroscopy
characterization of poly (vinyl alcohol) hydrogel with different hydrolysis
degree and chemically crosslinked with glutaraldehyde. Mater. Sci. Eng., C.

[52] Ahmadi
Bonakdar M., Rodrigue D. (2024). Electrospinning: Processes, structures,
and materials. Macromol..

[53] Uyar T., Besenbacher F. (2008). Electrospinning
of uniform polystyrene fibers: The
effect of solvent conductivity. Polymer.

[54] Tiwari S. K., Venkatraman S. S. (2012). Importance of viscosity parameters in electrospinning:
Of monolithic and core–shell fibers. Mater. Sci. Eng., C.

[55] Coelho S. C., Benaut P., Laget S., Estevinho B. N., Rocha F. (2022). Optimization of electrospinning parameters
for the production of
zein microstructures for food and biomedical applications. Micron.

[56] Lian S., Lamprou D., Zhao M. (2024). Electrospinning technologies
for
the delivery of biopharmaceuticals: Current status and future trends. Int. J. Pharm..

[57] Jaworek A. (2007). Micro-and
nanoparticle production by electrospraying. Powder Technol..

[58] Birer M., Acartürk F. (2022). Telmisartan loaded polycaprolactone/gelatin-based
electrospun
vascular scaffolds. Int. J. Polym. Mater. Polym.
Biomater.

[59] Yao C., Li X., Song T. (2007). Electrospinning and crosslinking
of zein nanofiber
mats. J. Appl. Polym. Sci..

[60] Wang M., Hai T., Feng Z., Yu D.-G., Yang Y., Annie Bligh S. (2019). The relationships
between the working fluids, process characteristics and products from
the modified coaxial electrospinning of zein. Polymers.

[61] Li X.-Y., Shi C.-J., Yu D.-G., Liao Y.-Z., Wang X. (2014). Electrospun
quercetin-loaded zein nanoribbons. Bio-Med.
Mater. Eng..

[62] Wen H.-F., Yang C., Yu D.-G., Li X.-Y., Zhang D.-F. (2016). Electrospun
zein nanoribbons for treatment of lead-contained wastewater. Chem. Eng. J..

[63] Malik R., Garg T., Goyal A. K., Rath G. (2015). Polymeric nanofibers:
targeted gastro-retentive drug delivery systems. J. Drug Target.

[64] Huang C., Soenen S. J., Van Gulck E., Vanham G., Rejman J., Van Calenbergh S., Vervaet C., Coenye T., Verstraelen H., Temmerman M. (2012). Electrospun cellulose acetate phthalate fibers
for semen induced anti-HIV vaginal drug delivery. Biomaterials.

[65] Tyo K. M., Vuong H. R., Malik D. A., Sims L. B., Alatassi H., Duan J., Watson W. H., Steinbach-Rankins J. M. (2017). Multipurpose
tenofovir disoproxil fumarate electrospun fibers for the prevention
of HIV-1 and HSV-2 infections in vitro. Int.
J. Pharm..

[66] Reddy V. S., Tian Y., Zhang C., Ye Z., Roy K., Chinnappan A., Ramakrishna S., Liu W., Ghosh R. (2021). A review on
electrospun nanofibers based advanced applications: From health care
to energy devices. Polymers.

[67] Jiang Q., Reddy N., Yang Y. (2010). Cytocompatible
cross-linking of electrospun
zein fibers for the development of water-stable tissue engineering
scaffolds. Acta Biomater.

[68] Wang S., Wang P., Liu S., Wang R., Li Y., Wang X., Ren F., Luo J., Fang B. (2024). Enhancement
of Mechanical Properties of Zein-Based Nanofibers by Incorporation
of Millet Gliadin. Foods.

[69] Vogt L., Liverani L., Roether J., Boccaccini A. (2018). Electrospun
Zein Fibers Incorporating Poly­(glycerol sebacate) for Soft Tissue
Engineering. Nanomaterials.

[70] Tuğcu-Demiröz F., Saar S., Kara A. A., Yıldız A., Tunçel E., Acartürk F. (2021). Development and characterization
of chitosan nanoparticles loaded nanofiber hybrid system for vaginal
controlled release of benzydamine. Eur. J. Pharm.
Sci..

[71] Dragar Č., Roškar R., Kocbek P. (2024). The incorporated drug
affects the properties of hydrophilic nanofibers. Nanomaterials.

[72] Chen R., Lin L., Wang H., Zhai X., Liang Y., Zhao B., Yu Z., Li K., Shen W. (2021). Effects of morphologies of thermosensitive
electrospun nanofibers on controllable drug release. Tissue Eng., Part A.

[73] Ilomuanya M. O., Bassey P. O., Ogundemuren D. A., Ubani-Ukoma U. N., Tsamis A., Fan Y., Michalakis K., Angsantikul P., Usman A., Amenaghawon A. N. (2023). Development
of mucoadhesive electrospun scaffolds for intravaginal delivery of
Lactobacilli spp., a tenside, and metronidazole for the management
of bacterial vaginosis. Pharmaceutics.

[74] Saar S., Demiröz F. N. T., Acarturk F. (2025). Development of Mucoadhesive Nanofiber
Platform Using Eudragit E100 for Vaginal Application. Hacettepe Univ. J. Fac. Pharm..

